# Small Community Water Systems Have the Highest Prevalence of Mn in Drinking Water in California, USA

**DOI:** 10.1021/acsestwater.3c00007

**Published:** 2023-05-08

**Authors:** Miranda L. Aiken, Samantha C. Ying

**Affiliations:** † Schmid College of Science and Technology, Chapman University, Orange, California 92866, United States; ‡ Environmental Sciences Department, University of California, Riverside, California 92521, United States; § Planetary Health Center, University of California Global Health Institute, San Francisco, California, 94158, United States; ⊥ School of Earth and Environmental Sciences, Schmid College of Science and Technology, Chapman University, Orange, California 92866, United States

**Keywords:** human right to water, water treatment, redox, groundwater, secondary contaminant

## Abstract

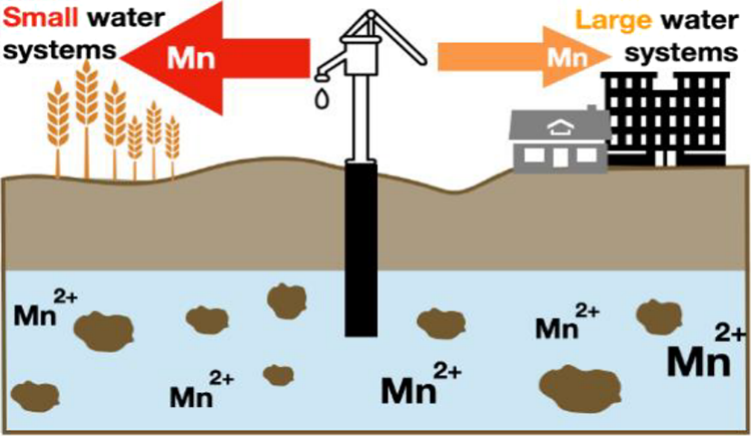

Manganese (Mn) is currently regulated as a secondary contaminant in California, USA; however, recent revisions of the World Health Organization drinking water guidelines have increased regulatory attention of Mn in drinking water due to increasing reports of neurotoxic effects in infants and children. In this study, Mn concentrations reported to California’s Safe Drinking Water Information System were used to estimate the potentially exposed population within California based on system size. We estimate that between 2011 and 2021, over 525,000 users in areas with reported Mn data are potentially exposed to Mn concentrations exceeding the WHO health-based guideline (80 μg L^–1^), and over 34,000 users are potentially exposed to Mn concentrations exceeding the U.S. Environmental Protection Agency health-advisory limit (300 μg L^–1^). Water treatment significantly decreased Mn concentrations compared to intake concentrations for all system sizes. However, smaller water systems have a wider range and a higher skew of Mn concentrations in finished water than larger systems. Additionally, higher Mn concentrations were found in systems above the maximum contaminant levels for chromium and arsenic. The treatment of these primary contaminants appears to also remove Mn. Lastly, data missingness remains a barrier to accurately assess public exposure to Mn in very small, small, and medium community water system-delivered water.

## Abstract 2

Over half a million users of community water systems in California are likely delivered water with Mn concentrations exceeding the new international health guidelines. Most of these systems are small and lack treatment infrastructure.

## 1 Introduction

Manganese (Mn) is a naturally occurring, redox-active mineral ubiquitous in soils, and sediments globally. Its release into groundwater is primarily due to microbially-mediated reductive dissolution of naturally occurring minerals controlled by local biogeochemical conditions.^1−3^ In surface waters, seasonal redox stratification can result in anoxic conditions favorable for the release of Mn in surface water;^4,5^ however, previous studies of Mn occurrence in surface and groundwater have demonstrated higher rates of exceedances in groundwater sources in the United States.^6^ Although less common, Mn from anthropogenic sources, such as industrial or mining activities, can be released into the environment^7^ or exacerbate its geochemical release.^8,9^

Extraction of drinking water from high Mn sources has previously been regarded only as an infrastructure challenge due to solid mineral deposition or aesthetic issues.^10,11^ However, recent research has linked Mn overexposure to neurotoxic impact; exposure to Mn in drinking water exceeding 100 μg L^–1^ has been linked to lower intelligence quotient (IQ) scores,^12−14^ increased the risk of attention-deficit hyperactivity disorder^15^ and decline in academic achievement.^16^ In addition, higher concentrations of Mn in groundwater (200 μg L^–1^) were associated with higher infant mortality rates.^17^

Currently, the United States Environmental Protection Agency (USEPA) has two guidelines for Mn: the secondary maximum contaminant level (SMCL; 50 μg L^–1^) and the health-advisory level (HAL; 300 μg L^–1^). The California State Water Resource Control Board (SWRCB) follows the SMCL and enforces a consumer notification limit of 500 μg L^–1^. In California, SMCLs are enforceable,^18^ and if in exceedance, require quarterly monitoring, reporting to SWRCB, and recommended treatment to increase consumer acceptance (22 CCR§64449). As of 2022, a California Senate Bill has been introduced that will require the Office of Environmental Health Hazard Assessment (OEHHA) to prepare and publish an assessment of the health-based risk of Mn in drinking water for the state board to then consider establishing a primary drinking water standard.^19^ In 2021, the World Health Organization (WHO) issued a provisional guideline value of 80 μg L^–1^ Mn in drinking water, five times lower than the guideline value recommended in the previous issue of drinking water guidelines.^20,21^ This revised guideline was based on cumulative evidence from epidemiological and animal-based studies indicating Mn neurotoxicity. The aim of the guideline is to be protective of vulnerable populations, especially bottle-fed infants at risk of high Mn consumption, through both drinking water and infant formula.^22^

In California, approximately 39 million users are served by public community water systems.^23^ These systems are responsible for the extraction, monitoring, treatment, and distribution of water to users. In 2012, the state passed AB 685, or the Human Right to Water, which clearly outlined the universal right to clean, reliable, and affordable drinking water for all community water system (CWS) users. Despite this legislation, many systems do not meet these standards.^24^

Currently, little is known about Mn in water delivered to California CWS users and whether Mn concentrations vary by water system size. The goal of our study is to use the best currently available data to (1) determine the concentration of Mn in source and delivered water in CWSs throughout California and the number of users delivered water with Mn exceeding threshold values, (2) investigate whether the removal of Mn by treatment varies by water system size, and (3) whether the presence of Mn positively correlates with the presences of other redox-sensitive contaminants to elucidate biogeochemical controls of Mn release into groundwater sources accessed for domestic use.

## 2 Materials and Methods

To best estimate the number of CWS users receiving water with high Mn in California, we integrated reported water quality parameters at point-of-entry, delineations defining those served by CWS, and estimates for the population within each system. The impact of treatment was characterized by comparing water quality parameters at intake versus point-of-entry. Further consideration of groundwater quality parameters, such as other primary contaminants and redox-sensitive constituents, were used as best estimates of subsurface conditions favorable for Mn release. A summary of data sources and the number of datapoints per data type included in our analyses is provided in Tables S1 and S2.

### 2.1 Data Sources

#### 2.1.1 Water Quality Data

Water quality for CWSs was estimated using reported data collected from Safe Drinking Water Information System (SDWIS) between 2011 and 2021.^25^ Manganese and other contaminant concentrations (arsenic, chromium, and nitrate) were downloaded in addition to other groundwater quality data (pH, hardness reported as CaCO_3_, sulfate, dissolved organic carbon [DOC], and iron). Information on how the data was collected is provided by the California State Water Resources Control Board’s website (https://www.waterboards.ca.gov/drinking_water/certlic/drinkingwater/documents/edtlibrary/data_dictionary.pdf).

The US Environmental Protection Agency Unregulated Contaminant Monitoring Rule (UCMR) requires large water systems (>10,000 users) to test for unregulated contaminants. Data from the fourth UCMR (UCMR4) included manganese data collected between 2018 and 2020. Manganese concentration data was downloaded from the US EPA website (https://www.epa.gov/dwucmr/occurrence-data-unregulated-contaminant-monitoring-rule).

The flow path data were accessed upon request from the Division of Drinking Water in August 2020. These data include the flow path of surface or groundwater sources into receiving sources, including treatment or distribution points. Data on the relative contribution of each flow source into the distribution point was not available.

#### 2.1.2 CWS Boundaries

A CWS is defined as a system providing water for human consumption with 15 or more service connections or serving 25 or more people daily for at least 60 days per year (defined by the California State Water Resources Control Board). CWS boundaries were taken from Tracking California Water System Service Areas Tool (Tracking California). The “active” status of the CWS was confirmed via SDWIS, wholesale systems were removed, and then the boundaries were cleaned. A total of 2851 active CWS boundaries were included in the final layer and were obtained in March 2022.^26^

Information regarding the federal water system type, water source type, population (including transient and residential population), service connections (including agricultural, commercial, institutional, residential, and combined), fee code designation, treatment plant class, and distribution system class was obtained from SDWIS accessed in April 2022 (Table S1).

CWSs were stratified by residential population into very small (<500 users), small (501–3300 users), medium (3301–10,000 users), large (10,001–100,000 users), and very large (100,000+ users) water systems based on US EPA designations.^27^ CWSs with no reported residential population or domestic service connections were excluded.

### 2.2 Data Handling

#### 2.2.1 Estimating Population Receiving Water with Elevated Mn via CWSs

Manganese, iron, arsenic, and chromium data from SDWIS were used to estimate the population delivered water with elevated levels of regulated contaminants from a CWS between 2011 and 2021. All inactive or proposed facilities were removed from the analysis. Systems with the classification of transient-noncommunity and non-public were excluded. Definitions of various user populations are defined by the State Waterboard (https://www.waterboards.ca.gov/drinking_water/certlic/drinkingwater/docs/class_dec_tree.pdf). Non-detects were calculated to be the reporting limit divided by the square root of two.^28,29^ The reporting levels varied from 0.5 to 40 μg L^–1^ depending on the method used, but over 97% of data was reported with a reporting limit of 20 μg L^–1^.

The reported constituent concentration was joined with flow path data and those with no reported flow path information were excluded. To estimate the constituent concentration at point-of-use, we retained the entry point values that flowed directly into the distribution systems.^30,31^ To account for higher frequency sampling when in exceedance, samples collected on the same day from the same location were averaged. A 10 year mean was calculated for each water system to allow comparison since reporting frequency was highly heterogeneous. A summary of data after each vetting step is available in Table S2.

The UCMR4 data was collected between 2018 and 2020 from active water facilities. The location within the distribution system was coded, and only data at entry point to distribution or within distribution was included. If more than one point-of-entry value was reported during the sampling period, the mean of the reported values was calculated and reported. Non-detects were calculated to be the reporting limit divided by the square root of two.^28,29^ All data had a reporting limit of 0.4 μg L^–1^.

Population data from SDWIS Public Water System Information was used to estimate population exposure to concentrations above threshold values. Transient (e.g., recreation area, highway, rest area, and hotel/motel) and non-transient (e.g., industrial/agricultural, medical facility, and school) populations were not included in population calculations. All data sorting was done in Excel or RStudio (version 2022.02.1).

### 2.3 Potentially Exposed Population

To account for multiple sources within a distribution system, we calculated the potentially exposed population (PEP) similar to Balazs et al.^30^ The total population served by each CWS was apportioned into five Mn exposure categories based on the proportion of entry points for that CWS with mean reported Mn concentrations falling within each category. The population assigned to each exposure category was then summed across all CWS to estimate the exposed population. For example, to calculate the PEP for small CWS, we used the following equation:
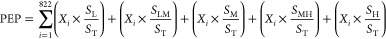
1where *X_i_* is the total population served by the CWS; *S* is the number of entry points for the CWS with a mean reported Mn concentration classified as low (*S*_L_), low-medium (*S*_LM_), medium (*S*_M_), medium-high (*S*_MH_), or high (*S*_H_); and *S*_T_ is the total number of point-of-entry sources for each CWS with reported data. For example, if a CWS was served by two point-of-entry samples with one sample classified as low and the other medium-high, half of the population served by this CWS would be classified as potentially exposed to low Mn and the other, medium-high Mn. Since no relative flow proportion for each sample was provided, it was assumed that the flow between each sample was equal.

### 2.4 Impact of Treatment Status on Constituent Concentration

The mean Mn concentrations at initial intake were compared to Mn concentrations at point-of-entry to determine whether and how much the water treatment decreased Mn concentrations as a function of CWS size. Possible water treatment(s) may include physical, biological, or chemical treatments and did not need to be targeted to treat Mn to be considered “treated” in our analysis. No specific treatment type designation was provided with reported values; therefore, the initial reported value in the flow path was considered as the intake concentration. If the initial reported value flowed into point-of-entry (i.e., only one value was reported), it was excluded from the analysis.

### 2.5 Geochemical Controls of Mn Release

The correlation between water quality parameters and reported Mn concentration were calculated to determine whether groundwater quality parameters (pH, hardness, sulfate, and Fe) and co-occurrence with other primary groundwater contaminants (As, Cr, and nitrate) varies with groundwater Mn. To account for potential temporal variation in sample collection, all data without an associated water quality parameter sample on the same date were excluded. The data were further separated by water source (groundwater or surface water) and treatment status (untreated or treated). All data sorting and vetting were done in Microsoft Excel or R (version 2022.02.1).

### 2.6 Statistical Analysis

Due to the non-normality of our data, non-parametric comparisons were used to test for significant differences in reported chemical data. The Kruskal–Wallis test with the Benjamini and Hochbert adjustment^32^ was used to determine significant differences in Mn concentration between CWS of different sizes (Table S5). The Mann–Whitney test was used to compare Mn concentration pre-and post-treatment (Table S6) and Mn concentration with co-occurring groundwater contaminant data above and below threshold values (Table S6). Spearman correlation analysis was also applied to examine the relationship between Mn concentration and various other reported water quality data (Tables S8 and S9). A conservative alpha level of 0.01 was used for each statistical test and all tests were performed in R (version 2022.02.1).

## 3 Results

### 3.1 Occurrence of Mn in Community Water Systems within California

**Figure 1:**
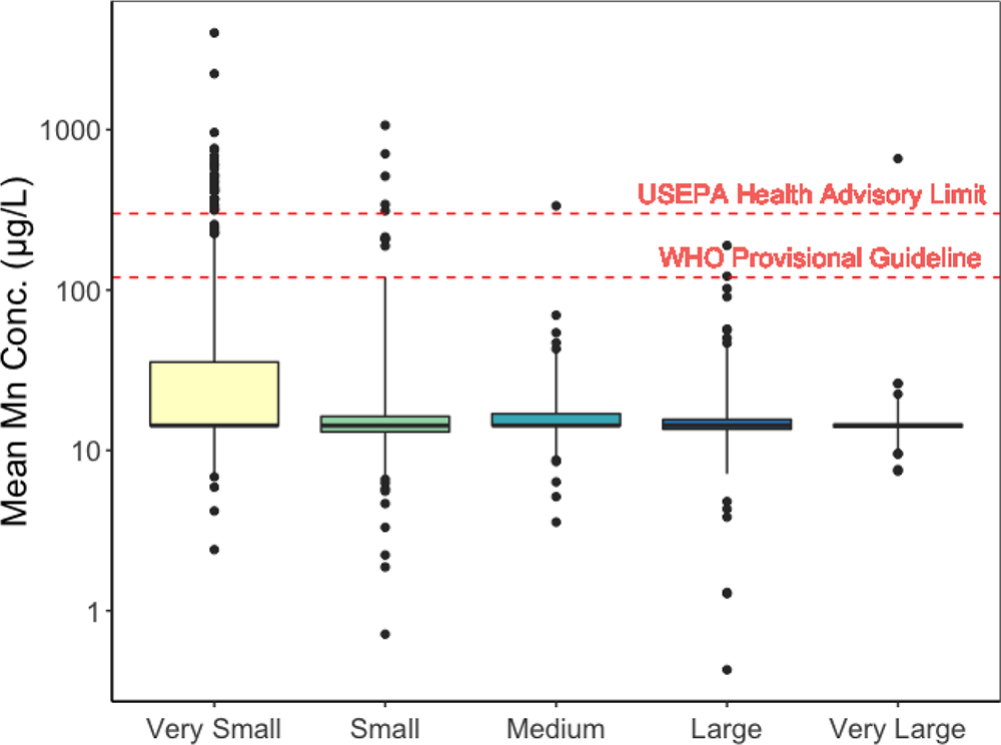
A ten-year mean Mn concentration measured at points leading directly into distribution systems calculated per year from 2011 to 2021. Boxes represent the 25th, 50th, and 75th percentile of concentrations and whiskers represent 5th and 95th percentiles. Outliers are represented as black points. A red dashed line is the USEPA Health-Advisory Limit for Mn (300 μg L^–1^) and WHO Provisional Guideline (120 μg L^–1^). No significant differences were observed (*p* < 0.01). Very small = <500 users (*n* = 823 CWS) , small = 501–3300 users (*n* = 170 CWS), medium = 3301–10,000 users (*n* = 86 CWS), large = 10,001–100,000 users (*n* = 151 CWS), very large = >100,000 users (*n* = 56 CWS).

From our analysis of reported Mn concentrations at CWS point-of-entry, over half a million CWS users are delivered drinking water with mean reported concentrations exceeding values that are linked to negative health impacts. A total of ∼39 million California residents rely on 2845 active CWS as their primary source of drinking water, 45% (1286) of which reported Mn concentrations at point-of-entry between 2011 and 2021. Over 61.4% of users are connected to very large CWS (>100,001 users) that report Mn concentration at point-of-entry more frequently than all other systems size classifications (Table 1). Consistently, very small systems have the largest percentage of the user population delivered water with higher reported Mn concentrations at point-of-entry, yet they serve 0.6% of the total user population (Table 1 and Figure 1). Overall, we estimate that at least 526,362 (1.4%) users within California between 2011 and 2021 were potentially exposed to Mn concentration in drinking water exceeding the WHO provisional guideline (80 μg L^–1^) and 34,460 (0.1%) exceeding the health-advisory limit (300 μg L^–1^). This is likely to be an underestimate since over a third (35.3%) of CWS within California did not report Mn concentrations at point-of-entry and were, therefore, not included in our estimates and analyses. No significant differences were observed between the median Mn concentration between the systems sizes; however, the interquartile range for very small systems was ∼10 times larger (21.2 μg L^–1^, Table S4) than the other systems (0.6–3.2 μg L^–1^, Table S4) and demonstrate larger variability is smaller systems.

**Table 1:** Total Population Served by a CWS with No Mn Data Reported and the Potentially Exposed Population (PEP)^b^ to Mn in Drinking Water^a^

	very small	small	medium	large	very large	total
total population	249,391	625,606	1,356,344	12,913,043	24,056,036	39,200,420
total population with no reported Mn at point-of-entry (count of water systems)	133,930 (905)	385,727 (294)	852,347 (134)	6,789,128 (193)	5,679,342 (33)	13,840,474 (1559)
total population with Mn data at point-of-entry (count of water systems)	115,461 (823)	239,879 (170)	503,997 (86)	6,123,915 (151)	18,376,694 (56)	25,359,946 (1286)
low: <50 μg L^–1^ (% of population)	93,238 (80.8%)	221,830 (92.5%)	479,399 (95.1%)	5,862,309 (95.7%)	17,986,247 (97.9%)	24,643,288 (97.2%)
low-medium: 50–80 μg L^–1^ (% of population)	4804 (4.2%)	5182 (2.2%)	18,197 (3.6%)	43,511 (0.7%)	118,992 (0.7%)	190,696 (0.8%)
medium: 80–120 μg L^–1^ (% of population)	3860 (3.3%)	7648 (3.2%)	0 (0%)	104,513 (1.7%)	235,373 (1.3%)	351,403 (1.4%)
medium-high: 120,300 μg L^–1^ (% of population)	9638 (8.3%)	1205 (0.5%)	0 (0%)	93,563 (1.5%)	36,083 (0.2%)	140,499 (0.6%)
high: >300 μg L^–1^ (% of population)	3921 (3.4%)	4014 (1.7%)	6500 (1.3%)	20,019 (0.3%)	0 (0%)	34,460 (0.1%)

a CWS size designation is as follows: very small (<500 users), small (501–3300 users), medium (3301–10,000 users), large (10,001–100,000 users), and very large (100,000+ users)

b Potentially exposed population (PEP) was calculated by multiplying the total CWS user population by the number of distribution point-of-entry falling within one of four Mn levels divided by the total number of distribution point-of-entries (Balazs et al. 2011).

From 2018 to 2020, the Environmental Protection Agency Fourth Unmonitored Contaminant Rule (UCMR4) required all community water systems serving over 10,000 people to report Mn concentration at entry points into the distribution system (EPA, 2022). All very large (>100,001 users) and 96.7% of large (10,000–100,000 users) systems reported Mn concentrations during UCMR4 (Table 2). Approximately 32,400 (0.08%) users were potentially exposed to Mn concentrations exceeding WHO provisional guideline (80 μg L^–1^) and 4060 (0.01%) exceeding the health-advisory limit (300 μg L^–1^, Table 2). Although this dataset reports Mn concentration at point-of-entry for 94.1% of the population, very small, small, and medium systems are severely underreported, which from previous analysis (Table 1), represents the population most likely to report Mn concentrations exceeding health guidelines.

**Table 2:** Total Population Served by a CWS with Mn Data Reported to 2018–2020 UCMR^a^

	very small	small	medium	large	very large	total
total population with no reported Mn (count of water systems)	247,639 (1718)	623,173 (460)	1,247,805 (200)	135,015 (11)	0 (0)	2,253,632 (2389)
total population with Mn data (count of water systems)	1246 (6)	18,158 (13)	130,622 (21)	12,921,656 (331)	23,832,558 (85)	36,904,240 (456)
low—<50 μg L^–1^ (% of population)	1246 (100%)	18,158 (100%)	120,599 (92%)	12,817,861 (99%)	23,832,558 (100%)	36,790,422 (99.7%)
low-medium—50–80 μg L^–1^ (% of population)	0 (0%)	0 (0%)	5963 (5%)	71,395 (1%)	0 (0%)	77,358 (0.2%)
medium—80–120 μg L^–1^ (% of population)	0 (0%)	0 (0%)	0 (0%)	32,400 (0%)	0 (0%)	32,400 (0.1%)
medium-high—120–300 μg L^–1^ (% of population)	0 (0%)	0 (0%)	4060 (3%)	0 (0%)	0 (0%)	4060 (0%)
high—> 300 μg L^–1^ (% of population)	0 (0%)	0 (0%)	0 (0%)	0 (0%)	0 (M)	0 (0%)

Most CWS are along the coastal region of California. Visual mapping of mean Mn concentration at intake and after available treatment at point-of-entry does not demonstrate any spatial trends in Mn occurrence at intake or after any available treatment (Figure S1).

### 3.2 Impact of Treatment on Mn at Point-of-Entry

**Figure 2:**
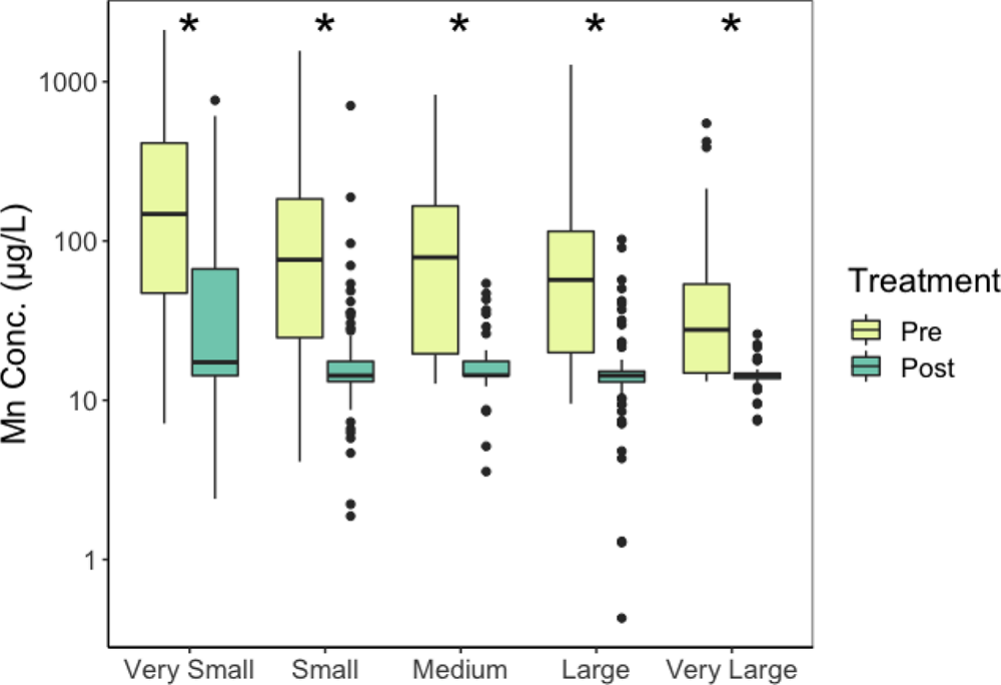
The impact of non-specific treatment on Mn concentration within CWS stratified by size. Pre-treatment is the 10 year mean of the reported values occurring first in the flow path and post-treatment is the 10 year mean of reported value at point-of-entry between 2011 and 2021. All data that did not have reported values for pre- or post-treatment are excluded from the analysis. The boxes represent the 25th, 50th, and 75th percentile of concentrations, and whiskers represent the 5th and 95th percentiles. Outliers are represented as black points. Very small = <500 users (*n* = 134 CWS) , small = 501–3300 users (*n* = 69 CWS), medium = 3301–10,000 users (*n* = 48 CWS), large = 10,001–100,000 users (*n* = 96 CWS), very large = >100,000 users (*n* = 50 CWS). Asterisks (*) indicate a significant difference in median pre- and post-treatment between each system size classification (*p* < 0.01, Table S6).

**Figure 3:**
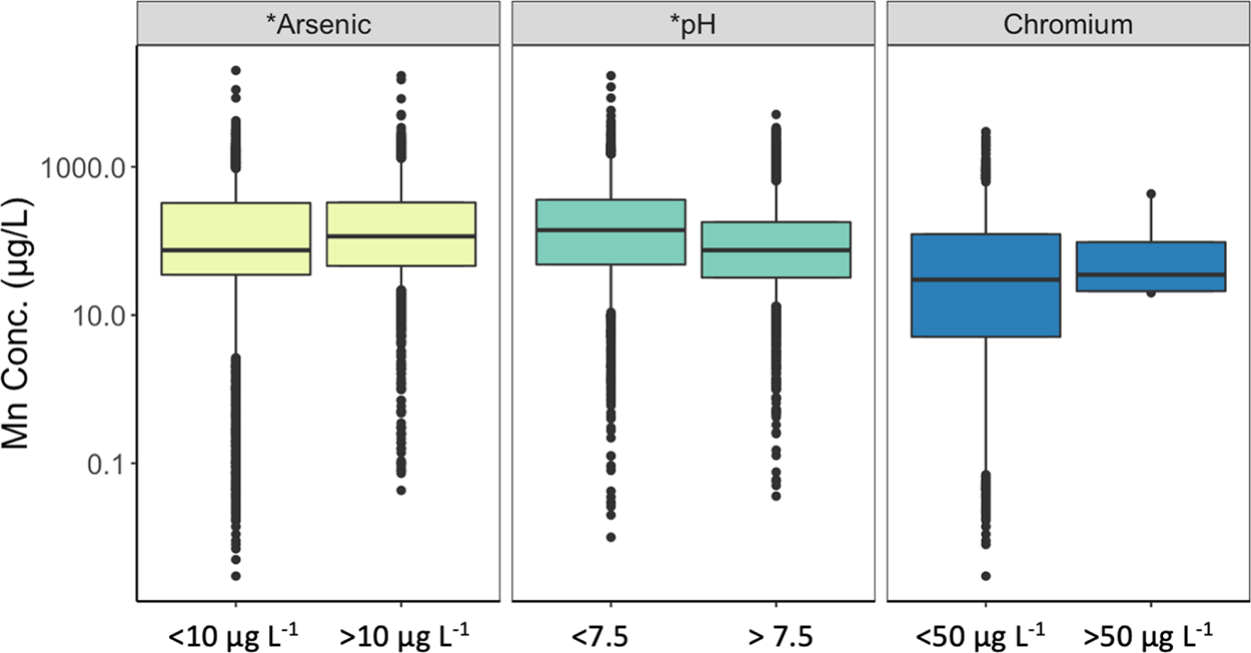
Raw groundwater Mn concentration in relation to primary groundwater contaminants As and Cr, and water pH from samples collected on the same day and sampling location. Boxes represent the 25th, 50th, and 75th percentile of concentrations, and whiskers represent the 5th and 95th percentiles. Outliers are represented as points. Asterisks (*) indicate significantly different median Mn values between concurrently measured co-contaminant or water quality values listed as categories at the bottom of the plot (*p* < 0.000, Table S7). The count of all samples analyzed is listed in Table S7.

The analysis of reported Mn data at intake versus point-of-entry revealed that the treatment of surface or groundwater following withdrawal greatly decreases Mn concentrations (Figures 2 and 3). The median concentration of Mn prior to treatment in small systems was 147.7 μg L^–1^ Mn but following treatment median Mn was 17.3 μg L^–1^, which is below the SMCL and WHO health-based guidelines. Although a smaller difference was observed in very large systems in comparison to smaller systems, a significant decrease was still observed between pre- (median of 27.7 μg L^–1^) and post-treatment (median of 14.3 μg L^–1^) Mn concentrations (Table S6).

### 3.3 Co-Occurrence of Mn with Other Contaminants

To better understand the co-occurrence of Mn with other contaminants, we analyzed As and Cr concentration data collected concurrently with Mn concentrations by CWS. We observed that raw groundwater extracted by CWS that exceeded California’s maximum contaminant level for As (10 μg L^–1^) are also likely to have higher median Mn concentrations than water with As below the MCL (Figure 3). The median Mn concentration for groundwater with As below the As MCL (75 μg L^–1^ Mn) was significantly lower than when As concentration exceeded the MCL (115 μg L^–1^, *p* < 0.000, Table S7) in untreated groundwater. However, a significant difference in Mn concentration in raw groundwater was not observed (Table S7, *p* = 0.29) when the maximum contaminant level for total Cr (50 μg L^–1^) was exceeded (Figure 3). Although statistically significant, total Cr (*r* = 0.08, *p* < 0.001) or As (*r* = 0.15, *p* < 0.001, Table S8) does not correlate with Mn in raw groundwater.

### 3.4 Co-Occurrence with Other Redox-Sensitive Groundwater Constituents

**Figure 4:**
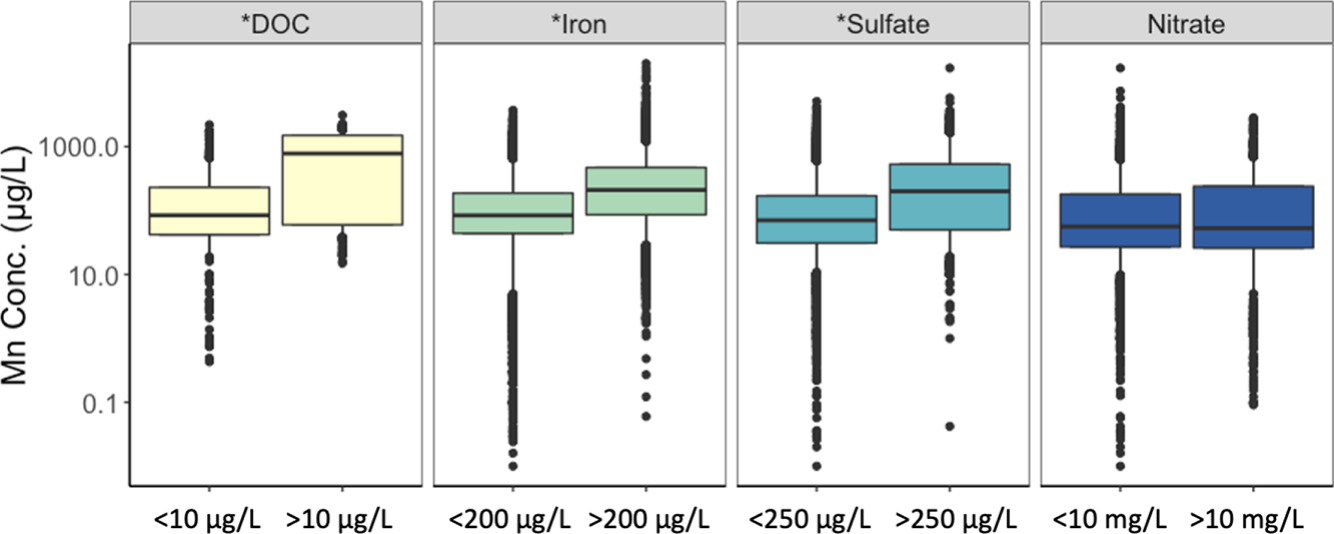
Raw groundwater Mn concentration in relation to other redox-sensitive groundwater constituents sampled on the same day and sampling location. Boxes represent the 25th, 50th, and 75th percentile of concentrations, and whiskers represent the 5th and 95th percentiles. Outliers are represented as points. * = significantly different median Mn values between concurrently measured co-contaminant or water quality values listed as categories at the bottom of the plot (*p* < 0.000, Table S7). The count of all samples analyzed is listed in Table S7.

Since Mn is a redox-sensitive groundwater contaminant, we also gathered all available ancillary chemical data for other redox-sensitive groundwater contaminants, including concentrations of nitrate, Fe, and sulfate, as well as dissolved organic carbon (DOC) concentrations, which fuels microbial metals reduction. A positive correlation was found between Mn and Fe (*r* = 0.43, *p* < 0.000) and Mn and DOC (*r* = 0.46, *p* < 0.000), whereas a slight positive correlation was observed with Mn and sulfate (*r* = 0.28, *p* < 0.000) in raw groundwater (Table S8). Following a similar pattern to the observed correlations, higher median Mn concentrations were observed in raw groundwater samples with Fe (SMCL = 200 μg L^–1^) and sulfate (SMCL = 250 mg L^–1^) exceeding SMCL values (Figure 4 and Table S7).

Additional chemical parameters that might influence Mn fate in groundwater, including pH and calcium carbonate (CaCO_3_), were also investigated. A slight positive correlation was observed between Mn and CaCO_3_ (*n* = 0.29, *p* < 0.000), whereas Mn was slightly negatively correlated with pH (*n* = −0.22, *p* < 0.000, Table S8).

## 4 Discussion

### 4.1 Mn Distribution and Reporting in CWS

From our analysis, we observed less reporting of Mn values at point-of-entry for very small (47.6% of systems reported) and small systems (36.3% of systems reported) than very large systems (62.9% of systems reported). However, all very large systems reported Mn at point-of-entry in the UCMR4, but only 0.3% of very small and 2.7% of small systems reported in this dataset, likely because reporting is only required for systems >10,000 users.^33^ Additionally, we found a higher percentage of very small system users (14.2%) potentially exposed to Mn concentrations exceeding the WHO guideline value at point-of-entry than very large systems (2.1%, Table 1). Previous analyses of primary contaminant violation in community water systems have also reported higher instances of primary contaminant violations in smaller systems and have attributed this difference to difficulty in accessing treatment options.^29,30,34−37^ Larger systems have better economy-of-scale and can easily distribute management and treatment costs across a large user base. In contrast, since small systems serve less users, the costs of additional treatment may no longer meet user affordability requirements.^38−40^

In an analysis of the USEPA’s UCMR dataset, which included monitoring of Mn in finished water of public systems, it was observed that 12.8% of public water systems exceeded 50 μg L^–1^ Mn and 2.1% of public water systems reported Mn concentrations exceeding 300 μg L^–1^.^6,33^

Here, we report that approximately 15.2% of CWS with reported 10 year mean Mn concentrations exceeded the SMCL (50 μg L^–1^ Mn) and 3.1% exceeded the HAL (300 μg L^–1^) in California systems, most of which were small or very small systems. This is similar to the national average, yet slightly higher due to the inclusion of more very small or small systems in comparison to larger systems. While smaller systems are required to be included in UCMR sampling events, only 800 small systems were included out of over 10,000 sampled systems.^33^ Since smaller systems may lack monitoring and treatment infrastructure due to the associated costs, their inclusion in large-scale monitoring programs, such as future UCMR sampling events, is critical to identify populations delivered water with high Mn or other contaminants.

The observed differences in the range of Mn concentrations at point of entry between CWS based on size can be attributed to the diminished technical, managerial, and financial (TMF) capacity of smaller systems, which limits consistent monitoring and adjustments in treatment. These disparities can be further exacerbated by a range of sociopolitical barriers, including proximity to polluting sources, lack of political power, and limited access to financial resources.^38^ Smaller systems often rely on fewer surface or groundwater intake points than larger systems, and if the source water violates water quality standards, they are often unable to switch to a different intake source.^41^ Drought conditions likely also exacerbate user affordability issues in smaller systems due to the depletion of long-term water storage.^42,43^

### 4.2 Mn and Impact of Treatment

Outside of the UCMR4 monitoring event, previous analysis of Mn exceedances in drinking has largely focused on untreated, raw groundwater concentrations,^3,44−50^ which are not representative of Mn concentrations at CWS point-of-entry. Our analyses show that Mn concentrations are significantly lower at point-of-entry than raw groundwater across all system sizes following treatment of any form (Figures 2 and 3). Common treatments used to remove Mn, include oxidation/precipitation, physical treatment, biological treatment, and infrastructure management. In larger systems that have more resources for treatment, Mn are often treated for aesthetic reasons since water with Mn concentrations greater than 50 μg L^–1^ can appear discolored or have a metallic taste.^51^

### 4.3 Co-Treatment of Primary Contaminants

Despite generally having less resources, very small and small systems Mn concentrations were significantly lowered following available treatment (Figure 2) likely due to required treatment of primary contaminants to meet state-wide, enforceable standards. For example, our results show that the median Mn concentration was higher in raw groundwater that also exceeded the MCL for As for all system sizes (Figure 3 and Table S10). Treatment of groundwater contaminated with As is required to meet state drinking water standards. Common treatments for As in groundwater is oxidation/filtration via ozone, chlorine dioxide, or other oxidants, and followed by membrane filtration, which will also result in the oxidation and removal of aqueous Mn.^52−54^ The treatment of other primary contaminants, such as nitrate, through processes, such as ion exchange, reverse osmosis, or electrodialysis, may also result in the removal of Mn.^55,56^ However, in our analysis and others,^48,57−59^ Mn and nitrate were not co-located in groundwater extracted for domestic use, most likely due to higher concentrations of nitrate observed in shallow, oxidizing conditions in close proximity to anthropogenic sources, and the presence of Mn in reducing conditions. Therefore, the systems away from anthropogenic sources with low nitrate may still have high concentrations of naturally occurring Mn. Thus, the nitrate does not act as a primary contaminant “indicator” for Mn.

The reporting of water treatment technologies applied for individual CWS is minimal, making it difficult to quantitatively assess whether specific treatments used to remove primary contaminants are sufficient for Mn removal in CWS of different sizes. Further inquiry at the individual CWS level for treatment technologies used and their Mn removal effectiveness is required to better understand the impact of primary contaminant treatments on Mn prior to distribution. Specific attention must also be paid to small systems where treatment may be prioritized for primary contaminants, but not for Mn.

### 4.4 Treatment Options and Feasibility

A common removal technique is to facilitate the oxidation of dissolved Mn(II) followed by the removal of the Mn(III/IV) particulates via filtration. Common oxidants used are oxygen, chlorine dioxide, ozone, and permanganate.^52,53^ It is well known that Mn(II) can precipitate in well-oxygenated water, however, the process is slow at circumneutral pH and may require a longer residence time or stronger oxidants to facilitate a quicker removal.^60^ Additional water quality parameters, such as DOC or reactive metals like Fe, that could inhibit Mn oxidation, must also be considered.^61^ Once the precipitation occurs, the suspended Mn(III/IV) oxides must be removed via filtration. Conventional media filtration is often sufficient to remove the suspended particles, but if direct oxidation results in the formation of ultrafine particles, membrane microfiltration or ultrafiltration may be required.^62^ Removal of Mn via filtration is also possible without prior chemical oxidation by the adsorption of Mn directly on the filtration media, such as greensand filters^63,64^ or direct ion exchange.^65^ However, there is evidence that greensand filters can result in Mn release into drinking water due to the dissolution of accumulated Mn or filter media if improperly used or maintained.^66^ Biofiltration, or filtration media that supports the growth of biofilms, has also been demonstrated to remove Mn in drinking water without any chemical additions. Three pathways of Mn removal are possible using this method: direct intracellular oxidation, extracellular adsorption, or oxidation by biofilms produced by microorganisms.^56^

Although the above methods are effective at Mn removal, various other water quality parameters, such as DOC, Fe, and dissolved oxygen, must also be considered when assessing removal effectiveness. Within our study, we observed higher concentrations of Mn co-occurring with higher concentration of DOC and Fe (Figure 4), which may limit the effective removal of Mn using these methods. Frequent monitoring not only of Mn, but of other water quality parameters, is required to ensure removal. Smaller systems that lack infrastructure support may not have access to consistent monitoring to determine if break-through is occurring prior to distribution.

Infrastructure management may also represent a path to minimizing Mn in delivered water. If the water system contains multiple wells, then it is possible to mix water from high Mn wells with a low Mn well prior to treatment or distribution to meet water quality standards. This management method, known as water blending, may be possible for larger water systems; however, is not feasible for small water systems that rely only on one intake source.^41^ The most direct and cost-effective method to reduce Mn consumption is to simply remove the well from use if it exceeds regulatory standards; again, this is only an option to systems drawing from more than one well.

Further management options, such as consolidation of small water systems with larger systems may also be a feasible mitigation solution. Approximately 66% of very small or small CWS are in close proximity (4.8 km) to larger systems where consolidation is considered a viable option.^67^ Consolidation will improve the economy-of-scale since smaller systems will now have access to the improved infrastructure and management provided to larger systems. In addition, the cost of increased monitoring and treatment will then be shared among a larger user base, which would increase water affordability. Despite evidence of the effectiveness of consolidation for water quality improvement,^41,68^ there are associated risks, such as loss of local autonomy and the large initial financial investment.^41^ Further allocation of the state-funding to support consolidation is required to enhance feasibility and ensure the effectiveness of this management method.^69^

Our findings showed that a higher percentage of very small and small systems exceeded the health-based threshold for Mn in drinking water than larger systems and targeted mitigation measures within these communities are needed. Further consideration of other factors that prevent access to infrastructure common within rural or disadvantaged communities, such as lack of political power or formally unincorporated communities,^67,68^ will also improve equitable distribution of state-sponsored funds.

### 4.5 Mn Geochemistry of Release

Manganese mobility in subsurface environments is predominantly controlled by biotic and abiotic redox transformations that result in either Mn immobilization through precipitation and adsorption reactions^1,70,71^ or mobilization via microbially-driven reductive dissolution during anaerobic respiration.^72,73^ We found that Mn in raw groundwater was positively correlated with Fe which is similar to findings in other studies.^3,45,50^ Although a less favorable electron acceptor than Mn, Fe is often observed in groundwater under similar conditions as Mn due to the overlap in redox potentials favorable for their reduction^2^ and the mixing of water from different zones during well screening.^74^

A factor driving the release of redox-sensitive contaminants is the presence of DOC. Groundwater with higher DOC often exhibits rapid depletion of available oxygen and nitrate due to microbial respiration, leading to reducing conditions favorable for the reductive dissolution of available Mn and Fe oxides.^3,74−76^ Therefore, the positive correlation observed between DOC and Mn in raw groundwater supports our conclusion that reductive dissolution of Mn minerals is a primary driver of Mn mobilization into groundwater accessed for domestic use. Higher concentrations of DOC near riverbanks or infiltrating surface water often drive nearby reducing zones and the release of Mn and Fe into groundwater.^3,77^

We observed no correlation between Mn and nitrate concentrations in raw groundwater sources reported by CWS. Periodic influxes of nitrate driven by infiltration or agricultural use can temporarily suppress or buffer Mn reduction since nitrate is more thermodynamically favored for microbial respiration.^75,78^ Elevated concentrations of Mn and Fe observed at depths where nitrate concentrations were low supports the hypothesis that Mn mobilization is redox controlled in this region.

The analysis of subsurface geochemical conditions favorable for Mn dissolution has demonstrated the formation of “hot-spots” for Mn release into groundwater,^1,47,58^ but this may occur at a spatial resolution unable to be captured in this analysis due to the spatial heterogeneity of CWS boundaries. Rosecrans et al.^47^ applied machine learning techniques to model redox conditions and dissolved Mn in California’s Central Valley. Regional characteristics, such as lateral position within the Valley, depth to water table, and portion of poorly drained soils were the most important predictor variables of Mn concentration within groundwater and are consistent with our understanding of hydrological processes governing anoxic locations within the subsurface that drive Mn release. However, no such model currently exists for the entire state of California, where other subsurface characteristics may govern Mn precipitation and dissolution.

## 5 Data Limitations

### 5.1 Other Water Type Users

State small systems (less than 14 service connections) and domestic well users were not considered in this study due to infrequent water quality reporting for these populations across the state. An estimated 1.3 million individuals within California rely on domestic wells as their main source of water.^36^ Since these systems lack regular reporting or treatment, raw groundwater chemistry is more likely representative of the composition at tap. In the previous analysis, Mn in groundwater accessed by domestic well communities and reported water quality in CWSs in California’s Central Valley, more domestic well users (0.4%) then community well users (0.05%) were accessing Mn concentrations exceeding the 300 μg L^–1^ health-advisory limit.^37^ Further analysis of Mn concentrations in domestic versus public wells throughout the United States, McMahon et al.^3^ observed more Mn concentrations exceeding the 300 μg L^–1^ health-advisory limit in domestic wells (7.2%) than public wells (5.2%) due to the impact of land surface-soil-aquifer connections on Mn release and well depth. However, the treatment of Mn was not taken into consideration within their study, which likely underestimated differences in exposure between public and private water users since we demonstrated that CWS treatment reduces Mn in extracted groundwater (Figure 3).

### 5.2 Changes in Mn Concentration from Point-of-Entry to Tap

In the current study, we relied on reported water quality at point-of-entry, or the point at which the water enters the distribution system. However, once in the distribution system, Mn concentration may be further modified prior to use. The residence time in the distribution system has been linked to lower concentration of Mn at the tap due to the precipitation of Mn by Mn(II)-oxidizing bacteria in biofilms or oxidation by residual chlorine and oxygen.^79−81^ Although these processes would generally reduce dissolved Mn concentrations, physical disruption of the biofilms or precipitates within pipes by hydraulic disturbances (flushing or change in flow) or changes in water chemistry (pH, sulfate, or temperature) can disrupt previously deposited and immobilized Mn inside pipes and cause a pulse of exposure at-tap.^81^ All of this may occur after Mn entry into the distribution system; unfortunately, these effects were unable to be captured within the current study due to a lack of at-tap water quality data availability. Future studies and public data collection should focus on at-tap water quality assessment to assess the impact of residence time within the distribution system and better estimate Mn exposure. Currently, lead is the only regulated constituent that requires monitoring at-tap, and getting a true assessment of Mn at-tap would require significant resources.

Aversion behavior may also limit exposure to Mn in drinking water. Particulate Mn lower than 50 μg L^–1^ is visually detectable in drinking water as having a brownish foggy appearance. However, dissolved Mn(II) does not have any visual deterrents and does not impart a metallic taste below 7500 μg L^–1^.^51^ Because of this users may unknowingly consume Mn concentrations exceeding the WHO provisional guideline or the health-advisory limit.^51^ If visually detected in tap water, users are more likely to rely on purchased water or further treat water prior to drinking, therefore, potentially reducing exposure. Further studies are needed to assess whether aversion behavior is a potential contribution to decreased Mn exposure, particularly in communities served by small systems and domestic well communities with limited treatment options.

## 6 Future Implications

Manganese is currently undergoing further consideration as a primary groundwater contaminant.^19^ Between 2019 and 2022, Mn was relisted as a groundwater contaminant by the WHO^21^ and received a maximum acceptable concentration in drinking water by Health Canada,^82^ and its status as a secondary contaminant is undergoing review within the state of California.^19^ As our perspective in the U.S. shifts from regarding Mn as a nuisance chemical to one of health concern, we need to simultaneously understand the magnitude of the issue through regular monitoring in all water systems, regardless of the size. Currently, the lack of reported Mn data at point-of-entry for over 1.5 million California residents prevents an accurate assessment of the magnitude of Mn contamination in drinking water.

Current treatment methods within large water systems have demonstrated effective removal of Mn prior to point-of-entry; however, increased attention needs to be given to smaller water systems without similar economies of scale. Providing funds for improved treatment within these smaller systems may not only help address existing water quality problems but also address issues with high concentrations of Mn, such as increased access to water treatment, consolidation of smaller systems, or more frequent monitoring. State recognition of Mn as a contaminant of concern will likely lead to the allocation of funding for all aforementioned efforts and therefore improve our understanding Mn in California CWSs and the potential impact on public health.

## Supporting Information Available

Publicly available data sources; count of available Mn data from SDWIS from 2011 to 2021 used to estimate potentially exposed population; count of water quality data from SDWIS from 2011 to 2021 used to characterize geochemical parameters favorable to Mn release into groundwater; results from Kruskal–Wallis test comparing median Mn concentration between different community water system sizes; results from Mann–Whitney statistical tests for pre- and post-treatment mean Mn concentration between 2011 and 2021; map of mean Mn concentration in California CWSs; results from Mann–Whitney statistical tests of Mn concentration in raw groundwater co-occurring with other groundwater constituents sampled at the same point and time; results from Spearman correlations of Mn and other co-occurring groundwater constituents in raw, untreated groundwater samples; results from Spearman correlations of Mn and other co-occurring groundwater constituents in treated groundwater samples; results from Mann–Whitney statistical tests of Mn concentration in raw groundwater co-occurring with high (>10 μg L^–1^ As) and low (<10 μg L^–1^ As) concentrations of arsenic sampled at the same point and time (PDF)
