# Supplementary material for: Small Community Water Systems Have the Highest Prevalence of Mn in Drinking Water in California, USA
Source: ACS ES T Water. 2023 May 8;3(8):2168–78. doi: 10.1021/acsestwater.3c00007 (PMC10425977; doi:10.1021/acsestwater.3c00007)
Supplement: Supplementary file 1 — ew3c00007_si_001.pdf [file ew3c00007_si_001.pdf]

Supplementary Information

Small community water systems have highest prevalence of Mn in drinking water in California, USA

Miranda L. Aiken[1] and Samantha C. Ying[1,2,3]*

[1] Schmid College of Science and Technology, Chapman University, Orange, CA 92866, USA
[2]Environmental Sciences Department, University of California, Riverside, CA 92521, USA
[3]Planetary Health Center, University of California Global Health Institute, San Francisco, CA, 94158, USA

*Corresponding Author: Samantha C. Ying, samying@ucr.edu

SI Contents

6 pages total including title page.

Page S2:
Table S1. Publicly available data sources.
Table S2. Count of available Mn data from SDWIS from 2011 to 2021 used to estimate potentially exposed population.
Table S3. Count of water quality data from SDWIS from 2011 to 2021 used to characterize geochemical parameters favorable to Mn release into groundwater.

Page S3:
Table S4. Results from Kruskal-Wallis test comparing median Mn concentration between different community water system sizes.
Table S5. Results from Mann-Whitney statistical tests for pre- and post-treatment mean Mn concentration between 2011-2021.

Page S4:
Figure S1: Map of mean Mn concentration in California CWSs.
Table S6. Results from Mann-Whitney statistical tests of Mn concentration in raw groundwater co-occurring with other groundwater constituents sampled at the same point and time

Page S5:
Table S7. Results from Spearman correlations of Mn and other co-occurring groundwater constituents in raw, untreated groundwater samples.
Table S8. Results from Spearman correlations of Mn and other co-occurring groundwater constituents in treated groundwater samples.

Page S6:
Table S9. Results from Mann-Whitney statistical tests of Mn concentration in raw groundwater co-occurring with high (>10 µg L^{-1} As) and low (<10 µg L^{-1} As) concentrations of arsenic sampled at the same point and time.

Table S1. Publicly available data sources.

Data Type	Link to data
Water quality data in CWSs	https://www.waterboards.ca.gov/drinking_water/certlic/drinkingwater/EDTlibrary.html
CWS boundary	https://drinkingwatertool.communitywatercenter.org/data/
CWS information and population served	https://data.ca.gov/dataset/drinking-water-public-water-system-information

Table S2. Count of available Mn data from SDWIS from 2011 to 2021 used to estimate potentially exposed population.

Data processing step	Total	Extra Small	Small	Medium	Large	Extra Large
Count of reported Mn for active CA CWSs between 2011-2021	177723	25460	21584	19663	58207	52809
Count of reported Mn for active CA CWSs at point-of-entry	58735	10804	8581	7246	20188	11916
Total active CA CWSs with reported Mn	2654	1623	408	201	316	106
Total active CA CWSs with reported Mn at point-of-entry	1284	822	170	86	150	56

Table S3. Count of water quality data from SDWIS from 2011 to 2021 used to characterize geochemical parameters favorable to Mn release into groundwater.

	Mn	Cr	NO_3	As	Fe	$CaCO_3$	pH	Sulfate	DOC
Count reported to SDWIS between 2011-2021*	203544	69314	553018	231241	188308	82511	85109	88941	48299
Count above detection limit	78138	45234	470429	150500	56909	82276	85109	84689	45234
Count reported as raw groundwater	50764	8661	268256	78867	34563	43988	45069	47701	5211
Count able to be joined with corresponding Mn by date and sample point	--	1338	5853	12546	25932	10115	9812	10381	1527

*Retained inactive and proposed systems

Table S4. Results from Kruskal-Wallis test comparing median Mn concentration between different community water system sizes. All other pairs were not significantly different ($p>0.05$).

Size Pairs	p value
Small - Very Small	0.036
Very Small - Large	0.036
Very Small - Very Large	0.050

Table S5. Results from Mann-Whitney statistical tests for pre- and post-treatment mean Mn concentration between 2011-2021.

	**Count**	**Median Pre-treatment**	**Median Post-treatment**	**p**
Very Small	134	147.7	17.3	0.000
Small	69	76.3	14.3	0.000
Medium	48	79.1	14.3	0.000
Large	96	57.1	14.3	0.000
Very Large	50	27.7	14.3	0.000

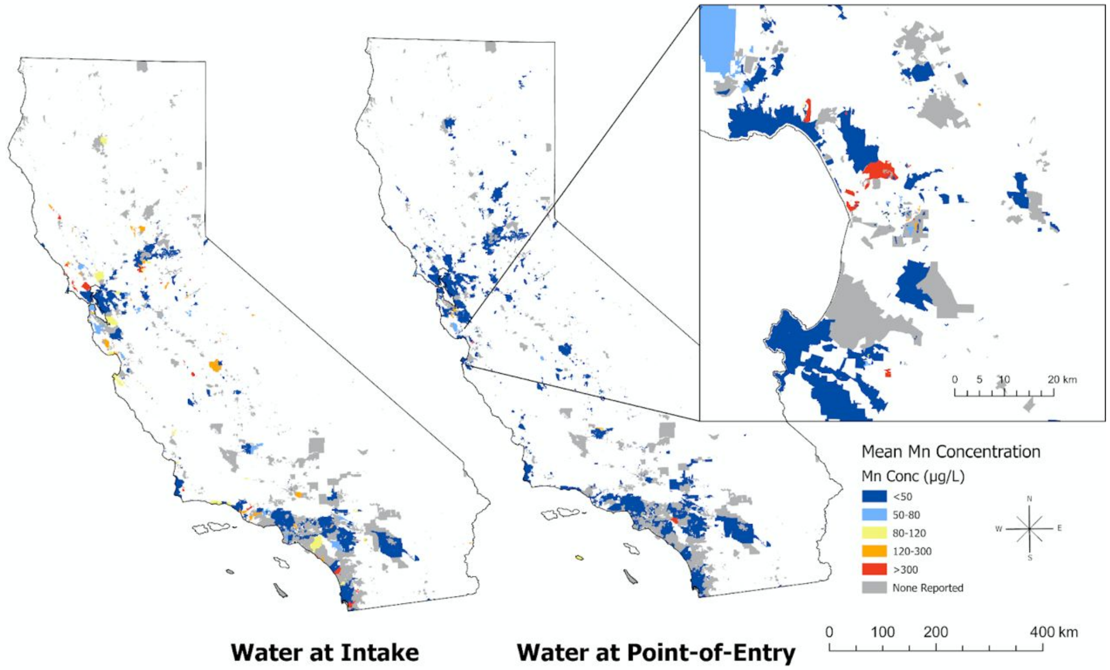

Figure S1. Mean Mn concentration in CWS between 2011-2021 at intake (left) and point-of-entry (right). Inlay zooms in on the Central Coast region to demonstrate that there is fine-scale heterogeneity in Mn contamination when considering very small and small community water systems.

Table S6. Results from Mann-Whitney statistical tests of Mn concentration in raw groundwater co-occurring with other groundwater constituents sampled at the same point and time. All samples below detection were removed from analysis. DOC=dissolved organic carbon. Cut-off values were captured from all listed MCL or SMCLs.

	Low	n	Median Mn	High	Median Mn	n	p-value
Mn-NO_3	NO_3 <10 mg L^{-1}	4433	56.1	NO_3 >10 mg L^{-1}	53	1421	0.45
Mn-Cr	Cr <50 µg L^{-1}	1329	26.7	Cr >50 µg L^{-1}	9	275	0.29
Mn-DOC	DOC <1.5 mg L^{-1}	701	84.3	DOC >1.5 mg L^{-1}	780	827	0.00
Mn-As	As <10 µg L^{-1}	6912	75	As >510 µg L^{-1}	115	5640	0.00
Mn-Fe	Fe <200 µg L^{-1}	11453	84	Fe >200 µg L^{-1}	210	14493	0.00
Mn-pH	pH <7.5	1090	140	pH>7.5	75	8733	0.00
Mn-Sulfate	Sulfate > 250 µg L^{-1}	6849	70.4	Sulfate > 250 µg L^{-1}	200	3534	0.00

Table S7. Results from Spearman correlations of Mn and other co-occurring groundwater constituents in raw, untreated groundwater samples.

Variables	n	p-values	Correlation Coefficient (r)
Mn-NO_3	5853	0.00	-0.06
Mn-Cr	1338	0.00	0.08
Mn-DOC	1527	0.00	0.49
Mn-As	12546	0.00	0.15
Mn-Fe	25932	0.00	0.43
Mn-pH	9812	0.00	-0.22
Mn-Sulfate	10381	0.00	0.28
Mn-$CaCO_3$	10115	0.00	0.29

Table S8. Results from Spearman correlations of Mn and other co-occurring groundwater constituents in treated groundwater samples.

Variables	n	p-values	Correlation Coefficient (r)
Mn-NO_3	222	0.00	-0.27
Mn-Cr	128	0.27	0.1
Mn-DOC	85	0.25	-0.13
Mn-As	1688	0.00	0.11
Mn-Fe	4319	0.00	0.74
Mn-pH	976	0.83	0.01
Mn-Sulfate	583	0.00	0.26
Mn-$CaCO_3$	705	0.49	-0.03

Table S9. Results from Mann-Whitney statistical tests of Mn concentration in raw groundwater co-occurring with high (>10 μg L^{-1} As) and low (<10 μg L^{-1} As) concentrations of arsenic sampled at the same point and time.

	Low Arsenic (<10 μg L^{-1})		High Arsenic (>10 μg L^{-1})		
CWS size	n	Median Mn (μg L^{-1})	n	Median Mn (μg L^{-1})	p
Very Small	1485	110	1534	170	0.00
Small	1040	68.5	1559	91.7	0.00
Medium	1000	569	805	250	0.00
Large	2569	58	1268	75.5	0.00
Very Large	753	40	394	81.4	0.00
